# A Narrative Review of Four Different New Techniques in Primary Anterior Cruciate Ligament Repair: “Back to the Future” or Another Trend?

**DOI:** 10.1186/s40798-018-0145-0

**Published:** 2018-08-09

**Authors:** Michael-Alexander Malahias, Dimitrios Chytas, Kaori Nakamura, Vasileios Raoulis, Masashi Yokota, Vasileios S. Nikolaou

**Affiliations:** 1 2nd Department of Orthopaedics, School of Medicine, National and Kapodistrian University of Athens, Athens, Greece; 2 Department of Joint Surgery and Sports Medicine, Graduate School of Medical Sciences, Tokyo Medical and Dental University, 1-5-45 Yushima, Bunkyo-ku, Tokyo, 113-8510 Japan; 3 Department of Orthopaedic Surgery, Faculty of Medicine, School of Health Sciences, University of Thessaly, Larissa, Greece; 4 Department of Sports Medicine, Hokkaido University Graduate School of Medicine, Sapporo, Japan; 5 ATOS Hospital, Heidelberg, Germany; 6 Orthopaedic Surgeon, ATOS Klinik, Schlossberg 21, 69117 Heidelberg, Germany

**Keywords:** Primary ACL repair, Dynamic intraligamentary stabilization, Bridge-enhanced ACL repair, Internal brace, Suture anchors, Literature review

## Abstract

Recently, four different operative techniques, referring to the primary anterior cruciate ligament (ACL) repair, were described. These are the dynamic intraligamentary stabilization (DIS) with Ligamys™, the Bridge-enhanced repair (BEAR), the use of internal brace, and the refixation with suture anchors. The purpose of this study was to assess the already-published, clinical, and pre-clinical results of those techniques. A literature review was conducted and implemented by three independent researchers. Inclusion criteria were clinical or cadaveric or animal studies about patients suffering from ACL rupture, who were treated with one of those four different arthroscopic techniques of primary ACL repair. There were 10 clinical trials dealing with the different techniques of primary ACL repair and 12 cadaveric or animal studies. The majority of the published clinical trials investigated the dynamic intraligamentary stabilization (DIS), while only four studies referred to the three other surgical techniques. Most of the clinical trials suggested that primary ACL repair should be done during the first 14–21 days after a proximal ACL rupture and not later. Further clinical evidence is needed for the techniques of bridge-enhanced ACL repair, internal brace, and suture anchors ACL refixation in order to support the animal and cadaveric biomechanical studies. Till now, the existing clinical trials were not enough to establish the use of those techniques in the ACL-ruptured patients. On the contrary, the Dynamic intraligamentary stabilization with Ligamys™ device demonstrated very promising results in different types of clinical studies.

## Key points

- The findings of this review suggest that orthopedic science has moved “back to the future” by rediscovering previous anterior cruciate ligament (ACL) repair strategies couched in sophisticated, modernized surgical techniques of the twentieth-first century.
- The dynamic intraligamentary stabilization (DIS) with Ligamys™, the Bridge-enhanced repair (BEAR), the use of internal brace, and the suture anchors are among these methods.
- The existing clinical trials are not enough to establish the use of (a) bridge-enhanced ACL repair, (b) internal brace, or (c) suture anchors ACL refixation for primary ACL repair. In contrast, the dynamic intraligamentary stabilization with Ligamys™ device has demonstrated very promising results in different types of clinical studies.

## Background

Between 34 and 44 out of 100,000 people in western countries undergo annually an anterior cruciate ligament (ACL) reconstruction, a fact that demonstrates the frequency of this type of injury in the general population [1, 2]. However, there is a controversy concerning the optimal management of ACL ruptures and several cases of inadequate recovery of knee function have been reported in the literature [3–5]. Indeed, rerupture often accompanies ACL reconstruction in young active adults when they return to sports [6–8]. Furthermore, in 10 to 30% of patients who reach their preoperative activity level after surgery, a second knee injury will follow [9–12]. Also, donor-site morbidity and weakening of secondary knee stabilizers have been reported as drawbacks of the use of autologous grafts, such as semitendinosus or patellar tendons [13–16].

According to Biau et al. [17], about 4 out of 10 patients recovered completely after ACL reconstruction; the percentages of a normal International Knee Documentation Committee (IKDC) score in the patients who underwent an operation with a semitendinosus or a ligamentum patellae graft were 33 and 41%, respectively. This fact could be possibly explained by the loss of the important role that native ACL plays in proprioception, due to the sensory nerve fibers which it contains [18, 19].

Apart from the negative influence of the ACL tear to the function of the knee, this injury has also been associated with the development of osteoarthritis [20]. So, a self-healing of a ruptured ACL would be beneficial for the proprioception and function of the knee joint; indeed, some studies have supported the possibility of biological self-healing of the ligament [21–23]. ACL transplants have shown inferior outcomes in comparison with the healthy ACL in terms of rupture rates, a fact that jeopardizes the stability of the knee joint after ACL reconstruction [8].

These factors have led the research to the exploration of techniques of arthroscopic primary ACL repair [24]. Historically, open primary ACL repair was the gold standard treatment in the 1970s and 1980s, demonstrating rather controversial results [25]. After the arrival of the knee arthroscopy and the establishment of the ACL reconstruction with grafts, this technique was abandoned [25, 26]. However, these evolutions are sometimes not linear and periodically undergo paradigm shifts [25]. Surprisingly, in the last 10 years, we noticed the development of various medical devices by different companies concerning an arthroscopic primary repair of the native ACL [27–30]. In a way the orthopedic research rediscovered the old primary ACL repair, but more sophisticated, as it is required nowadays, with the use of bioactive scaffolds, specialized devices, and arthroscopic minimally invasive techniques.

The dynamic intraligamentary stabilization (DIS) with Ligamys™ [31], the Bridge-enhanced repair (BEAR) [32], the use of internal brace [33], and the suture anchors [34] are among these methods. A number of pre-clinical [29, 30, 35–37] and clinical [38–43] studies investigated the efficacy of those techniques. A literature review was performed regarding the clinical and biomechanical results of the aforementioned operative techniques.

## Main text

### Materials and Methods

A narrative review of the literature was conducted by three independent reviewers (MM, DC, VR) who used the MEDLINE/PubMed database and the Cochrane Database of Systematic Reviews. These databases were searched using the terms “dynamic intraligamentary stabilization acl” and “bridge-enhanced acl repair” and “internal brace acl” and “anchors primary acl repair”. To maximize the search, backward chaining of reference lists from retrieved papers was also undertaken. As for the search about the term “dynamic intraligamentary stabilization acl”, from the 14 initial studies, we finally chose and assessed 6 clinical studies which were eligible to our inclusion-exclusion criteria plus 4 cadaveric or animal studies. Regarding the search of the term “bridge-enhanced acl repair”, from the 7 initial studies we finally chose and assessed 1 clinical comparative trial and 6 animal studies which were eligible to our inclusion-exclusion criteria. Furthermore, from the initial 34 articles which were found through the search of the term “internal brace acl,” only 1 clinical and 1 animal study were fitting to our criteria. Finally, from the 8 articles identified through the search of the term “anchors primary acl repair,” we chose 2 clinical and 1 animal study which met our criteria.

Inclusion criteria were clinical or cadaveric or animal studies dealing with a primary ACL repair technique (dynamic intraligamentary stabilization or bridge-enhanced ACL repair or ACL internal brace or suture anchors primary ACL repair). The clinical trials should have contained a clinical follow-up evaluation (with tests and/or scores), while all of the studies included must have been written in English or German. Furthermore, they should have been published by August 31, 2017 (end of our search).

Exclusion criteria were studies dealing with ACL reconstruction, studies not investigating arthroscopic techniques of primary ACL repair (dynamic intraligamentary stabilization or bridge-enhanced ACL repair or ACL internal brace or suture anchors primary ACL repair), articles not written in English or German, clinical trials not containing a clinical follow-up evaluation (with tests and/or scores), editorial comments, case reports, literature or systematic reviews or meta-analyses, duplicate studies, and articles written after August 31, 2017.

As a result, we excluded all the irrelevant studies, non-describing the four aforementioned techniques (27), technical notes (3), editorial comments (2), case reports (1), and articles with no clinical outcome (2). We did not include also systematic reviews (5) or meta-analyses. In addition, we excluded one pilot study of Eggli et al. which illustrated the short-term results (24 months) of a small series treated with DIS [43]. This study was fully overlapped by a study published 1 year later with the same patients, who were followed-up for a much longer period (60 months) [39]. Among these two studies, we chose to include in our review the later published with the long-term results [39].

**Fig. 1:**
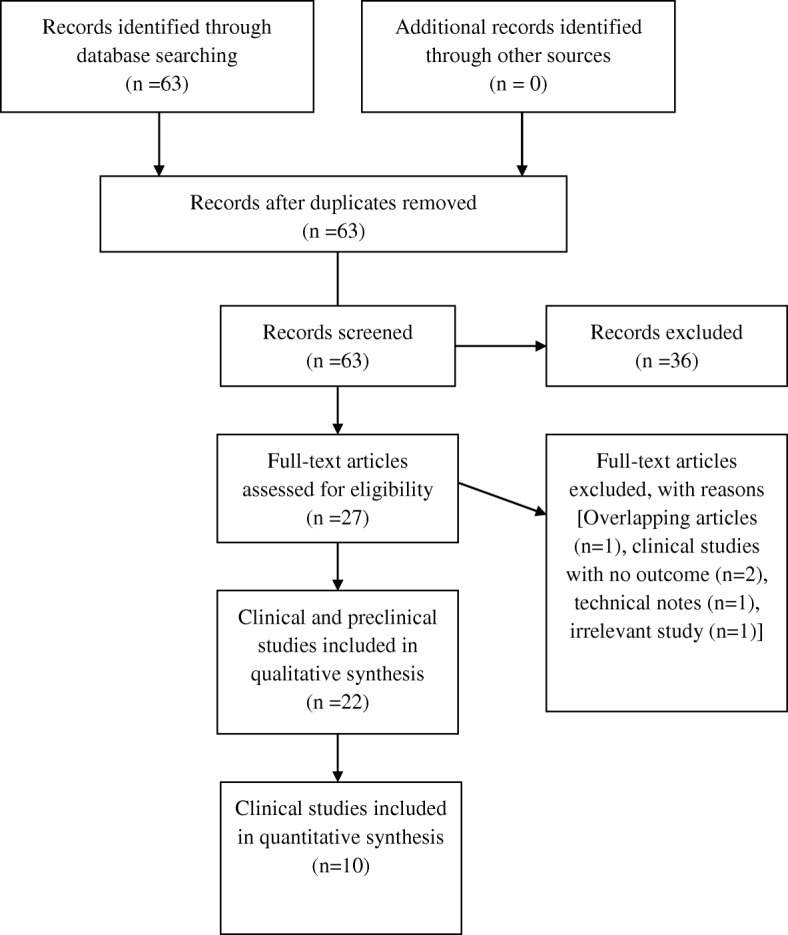
Flow chart of study selection according to PRISMA guidelines for reporting systematic reviews and meta-analyses

A summary flowchart of our literature search can be found in Fig. 1. The quality of the evidence was classified using the US Preventive Services Task Force system for ranking level of evidence.

Differences between reviewers were discussed until agreement was achieved. If no agreement could be reached, it was planned that the senior author (VN) would decide. The three reviewers (MM, DC, VR) independently extracted data from each study and assessed variable reporting of outcome data. The methodological quality of each study included and the detected bias were assessed independently by each reviewer. As for the clinical studies, the primary outcome measure was the postoperative statistically significant improvement of the clinical scores used in comparison with the preoperative scores per study. Secondary outcome was the reoperations’ rate per study. All data generated or analyzed during this study are included in this published article.

### Results

Overall, our search resulted in 10 clinical and 12 cadaveric and animal studies. From the clinical studies, 6 were about DIS, 1 about BEAR, 1 about internal brace, and 2 about anchors primary ACL repair (Table 1).

**Table 1:** Clinical studies: level of evidence and the type of the implant

Study	Year of publication	Technique	Level of evidence
Bieri et al. [31]	2017	DIS	III
Büchler et al. [38]	2016	DIS	IV
Eggli et al. [39]	2016	DIS	IV
Evangelopoulos et al. [40]	2017	DIS	III
Kösters et al. [41]	2015	DIS	IV
Henle et al. [20]	2015	DIS	IV
Murray et al. [32]	2016	BEAR	II
Smith et al. [33]	2016	Internal brace	IV
Achtnich et al. [34]	2016	Suture anchors	III
DiFelice et al. [42]	2015	Suture anchors	IV

Level of evidence II: individual cohort study or non-randomized, prospective, controlled, clinical study

Level of evidence III: case-control study

Level of evidence IV: case series

*DIS* dynamic intraligamentary stabilization, *BEAR* bridge-enhanced ACL repair

**Fig. 2:**
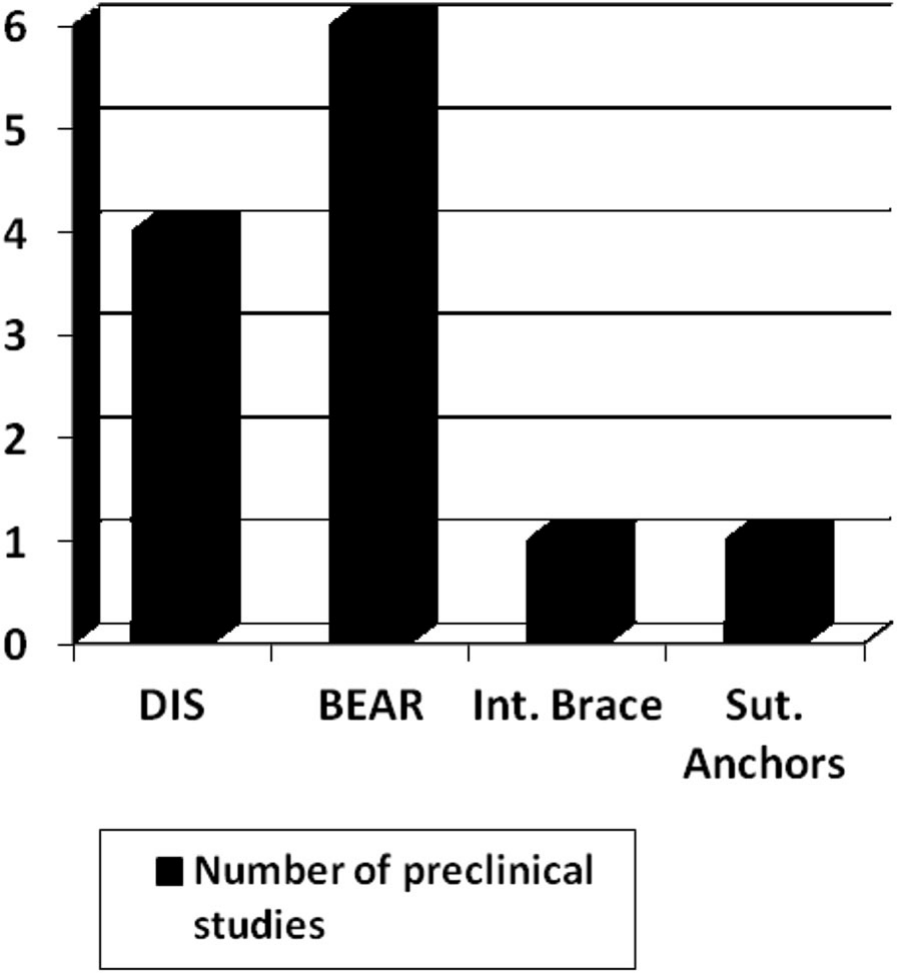
Bar graph depicting the number of preclinical studies investigating the DIS technique, the BEAR, the ACL internal brace and the suture anchors ACL repair. DIS: dynamic intraligamentary stabilization, BEAR: bridge-enhanced ACL repair, Int. Brace: internal brace, Sut. Anchors: suture anchors, ACL: anterior cruciate ligament

From the cadaveric and animal studies, 4 were about DIS, 6 about BEAR, 1 about internal brace, and 1 about anchors primary ACL repair (Fig. 2).

In total, there were 497 ACL-ruptured patients treated with the DIS, while 32 patients were operated with suture anchors, 10 patients with the BEAR technique and only 3 patients were treated with the use of the internal brace. The demographic characteristics of each clinical study are mentioned in Table 2.

**Table 2:** Demographic data and mean follow-up per clinical study

Study	Technique	Number of patients	Sex	Mean age (years)	Follow-up (months)
Bieri et al. [31]	DIS	53-DIS 53 Conventional reconstruction	43M:10F Per group	30 (1st group) 31 (2nd group)	24
Büchler et al. [38]	DIS	45	32M-13F	26	12
Eggli et al. [39]	DIS	10	8M-2F	23.3	60
Evangelopoulos et al. [40]	DIS	23 With collagen application, 33 without collagen application	15M-8F (1st group) 24M-9F (2nd group)	30 (1st group) 27 (2nd group)	24
Kösters et al. [41]	DIS	55	31M-24F	30.4	12
Henle et al. [20]	DIS	278	163M-115F	31	24
Murray et al. [32]	BEAR	20 (2 groups of 10)	4M-6F (BEAR) 2M-8F (Control)	24.1 (BEAR) 24.6 (Control)	3
Smith et al. [33]	Internal brace	3	1M-2F	6	12 21 24
Achtnich et al. [34]	Anchors primary ACL repair	21 (Anchors primary ACL repair)-20 (control)	No significant difference between sexes	30 (Anchors primary ACL repair) 33.6 (Control)	28
DiFelice et al. [42]	Anchors primary ACL repair	11	10M-1F	37	42

*DIS* dynamic intraligamentary stabilization, *BEAR* bridge-enhanced ACL repair, *M* males, *F* females, *ACL* anterior cruciate ligament

#### Clinical Studies

##### Dynamic Intraligamentary Stabilization (DIS)

**Fig. 3:**
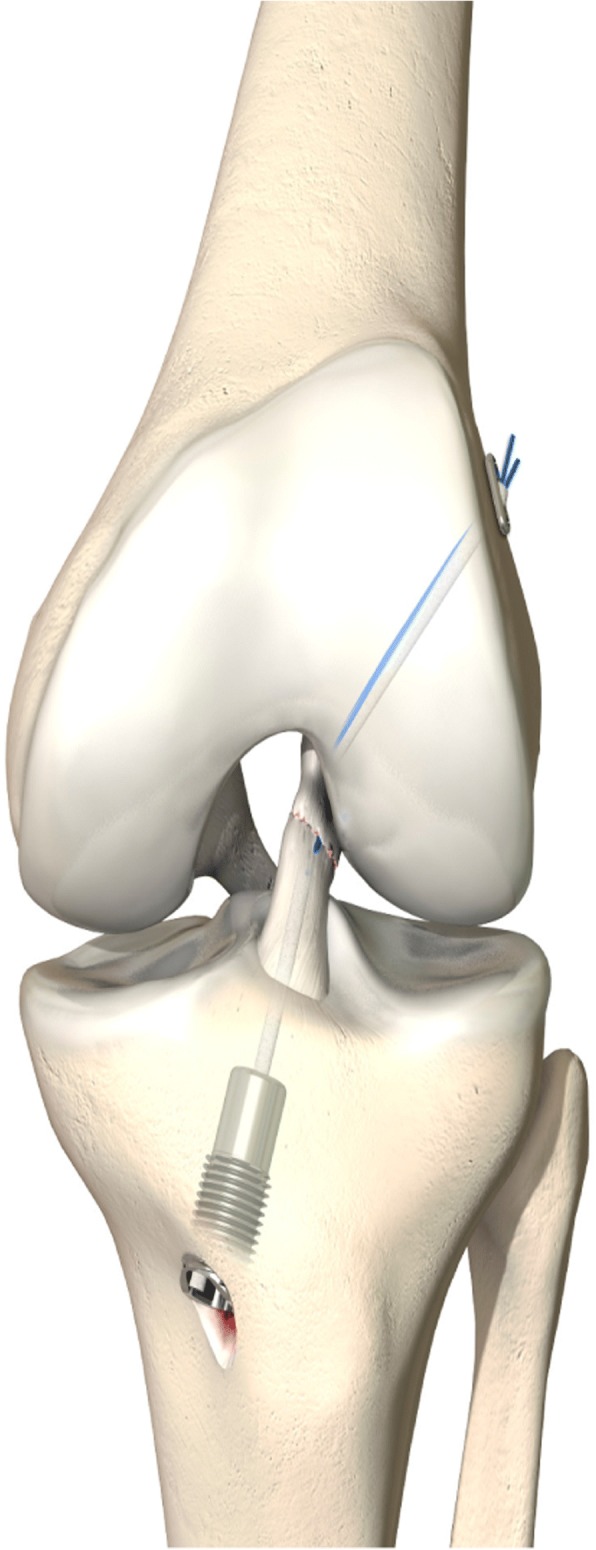
Dynamic intraligamentary stabilization (DIS) with Ligamys™ device (figure supplied by and reproduced with permission from MATHYS European Orthopaedics)

The DIS technique includes the insertion of a threaded sleeve from the anteromedial side of the tibia, with a preloaded spring and a mechanism for stabilizing the spring on the tibia (Fig. 3).

Afterwards, a braided wire is passed through the middle of the torn ACL and is secured to the lateral surface of the femur with a button. The knee is held in a posterior drawer by the preloaded spring, via which the two ends of the ruptured ACL are approximated [27].

Biery et al. found, after a 2-year follow-up that patients who underwent Dynamic intraligamentary stabilization (DIS) with Ligamys™ device (MATHYS European Orthopaedics) returned to work 1 month earlier than patients who underwent conventional anterior cruciate ligament reconstruction (ACLR) [31].This difference probably related to the earlier surgical timing of DIS in comparison with ACLR [31]. However, no difference was noted between the two groups regarding treatment costs, revision rates, and secondary arthroscopies. DIS, according to the authors, could be an acceptable treatment option for ACL rupture [31].

Furthermore, Büchler et al. studied patients who were operated with DIS technique and noticed, after a one-year follow-up, that the rerupture rate was low and, moreover, the functional recovery was satisfactory [38]. Also, Eggli et al., after a 5-year follow up of patients to whom DIS was performed for a fresh ACL rupture (up to 14 days), remarked that the survival rate was 80% [39]. The authors also underlined the excellent outcomes and satisfaction of patients with a functionally healed ACL at the last follow-up [39].

Some other authors evaluated the use of DIS with additional techniques for the amelioration of clinical outcomes. For example, Evangelopoulos et al. evaluated the application of a collagen membrane on the ACL of patients who underwent DIS with Ligamys™ device for a mid-substance ACL rupture and compared the outcomes with those of patients to whom a solitary DIS was performed [40]. After a 2-year follow-up, the authors found that in the former group of patients there was a reduced incidence of complications, such as reruptures and extension deficit [40]. So, it was concluded that solitary DIS for mid-substance ACL ruptures was an inferior treatment option to the additional application of a collagen membrane on ACL [40]. Moreover, Kösters et al. showed that DIS accompanied by microfracturing of the notch could biomechanically and biologically promote self-healing of a ruptured ACL [41].

In addition, Henle et al. performed a study which evaluated the outcomes of DIS at a series of patients, whose follow-up was 2 years [20]. The authors concluded that anatomical repositioning, combined with DIS and microfracturing, resulted in clinically stable healing of the ruptured ACL in most of their patients [20].

##### Bridge-Enhanced ACL Repair (BEAR)

Regarding the BEAR procedure [32], a No. 2 Vicryl suture is placed in the tibial end of the ruptured ACL and passes through a button with two No. 2 Ethibond sutures. The button passes through a drilled femoral tunnel and is secured on the lateral surface of the femur. The two looped Ethibond sutures (4 matched ends) are passed through a scaffold, which includes extracellular matrix proteins from bovine tissue, and through a drilled tibial tunnel. Ten milliliters of autologous vein blood are added to the scaffold, which is then passed into the femoral notch and the Ethibond sutures are tied over another button on the tibia. The ends of the No. 2 Vicryl suture are tied over the femoral button.

Murray et al. investigated whether bridge-enhanced anterior cruciate ligament (ACL) repair (BEAR) was associated with a deep joint infection (arthrocentesis with positive culture) or significant inflammation (clinical symptoms justifying arthrocentesis but negative culture) [32]. The authors found that BEAR procedure (Boston Children’s Hospital) did not cause such complications and, moreover, led to significantly better hamstring strength in comparison with ACL reconstruction with hamstring autograft, at a 3-month follow-up [32]. Effusion, pain, and failures did not differ significantly between the two groups of patients who underwent the two procedures [32].

##### Internal Brace

As far as the internal brace is concerned [33], a non-absorbable braided suture is put via a suture passing device through the proximal end of the torn ACL. The internal brace comprises a non-absorbable braided tape which is placed on a fixation device. The construct is pulled through the drilled tibial tunnel and, afterwards, up into the femoral tunnel via the passing sutures. The fixation device is put onto the lateral surface of the femur. The distal end of the internal brace is placed in the tibial metaphysis via a bioabsorbable suture anchor screw.

Our search resulted in one clinical study [33] and a case report, which was excluded from our analysis [44]. Smith et al. investigated the outcomes of ACL repair with the use of an internal brace (Arthrex) in a small series of three children [33]. Two children with a complete proximal ACL rupture and a third with a concomitant tibial spine avulsion underwent direct surgical repair, enhanced with an internal brace, which was removed after 3 months [33]. The authors noted that this method led to complete ACL healing and knee stability 3 months after surgery, a return to normal activities was done at 4 months, and excellent knee function without growth disturbance was found beyond 2 years [33]. The study demonstrated that internal brace could be an attractive alternative to ACL reconstruction, where an adequate ACL remnant allows direct repair [33].

##### Anchors Primary ACL Repair

According to the suture anchors technique, a twist-in cannula is put into the anteromedial portal [34]. Afterwards, a curved 90° Suture Lasso device is passed through the proximal end of the torn ACL. A wire loop is inserted through an anterolateral portal. Via the wire loop, a No. 2 Fiber Wire suture is shuttled through the end of the torn ACL. The hole for the anchor is drilled and the anchor is put in the middle of the ACL insertion. The ACL remnant is then reduced to the point of the femoral insertion of the ACL, where microfracture holes are made.

Two clinical studies were found after our search, regarding anchors primary ACL repair. Achtnich et al. compared the functional outcomes between two groups of patients who underwent ACL repair and reconstruction respectively, with a mean follow-up of 28 months [34]. The authors found that ACL repair (proximal refixation with knotless suture anchors and microfracturing, Arthrex) provided comparable results to ACL reconstruction. Furthermore, DiFelice et al. explored the clinical outcomes of arthroscopic suture anchor primary ACL preservation at a mean follow-up of 3.5 years and concluded that this method could be successful in carefully selected patients with excellent tissue quality and proximal avulsion ACL tears [42].

#### Animal and Cadaveric Studies

##### Dynamic Intraligamentary Stabilization (DIS)

Häberli et al. performed a biomechanical study to evaluate the course of translation during a simulated early postoperative phase after dynamic augmentation of anterior cruciate ligament tears [45]. The authors found a low increase in anteroposterior translation of the knees that were investigated and noted that this procedure supported ACL repair during biological healing. Moreover, Schliemann et al. investigated DIS from a biomechanical point of view and concluded that it could lead to a stable knee during ACL healing [35]. It was remarked that this technique was biomechanically superior to other methods of operative treatment of ACL rupture [35].

Also, Kohl et al. evaluated biomechanically the DIS technique and concluded that it resulted in close contact between the two ends of the torn ACL, so they concluded that this method could create an appropriate environment for healing of ACL and supply sufficient anteroposterior stability of the knee [27]. Finally, another study demonstrated the beneficial impact of DIS technique on self-healing of the torn ACL [8].

##### Bridge-Enhanced ACL Repair (BEAR)

Biercevicz et al. considered that their study concerning an animal model could significantly contribute to the development of a non-invasive technique to predict microscopic healing for BEAR method [36]. Moreover, Kiapour et al., after performing an animal study, concluded that the use of absorbable sutures during BEAR technique was associated with significantly inferior biomechanical outcomes in females to those in males, a difference which was not remarked when non-absorbable sutures were used [24]. Most recently, Kiapour et al. showed in a comparative animal study that novel ACL injury treatments, like the BEAR technique, can restore the structural and anatomic properties of the torn ACL to those of the native ACL in an effort to minimize the risk of early-onset posttraumatic osteoarthritis [46].

Furthermore, Proffen et al. evaluated, with an animal study, the use of electron beam irradiation for sterilization of extracellular matrix scaffolds for the reinforcement of ACL repair [37]. The authors found that this method of sterilization was effective and had no significant influence on in vivo function of the scaffolds. Moreover, Proffen et al. after an animal study showed that low-temperature ethylene oxide and electron beam irradiation were acceptable methods of sterilization of reconstituted extracellular matrix-derived scaffolds [28]. The slight modification of in vitro scaffold properties did not significantly alter biologic reactions of the surrounding tissues in vivo.

Finally, Fleming et al. investigated in animals the use of an extracellular matrix scaffold combined with platelets to reinforce healing of an ACL graft [47]. The authors demonstrated that only the 1× platelet concentration ameliorated healing for traditional ACL reconstruction, the positive results were not enhanced by the use of 5× platelet concentration, while a decreased cartilage damage after ACL reconstruction was noticed with the use of platelets.

##### Internal Brace

Our search resulted in only one animal study about internal brace, while no cadaveric studies were found. The aim of this study, by Cook et al., was to describe a canine model for all-inside arthroscopic complete ACL reconstruction, with the use of a quadriceps tendon allograft with internal brace (QTIB) [29]. Ten animals underwent complete transection of the native ACL followed by all-inside ACL reconstruction with the QTIB construct with suspensory fixation, while 10 contralateral knees were used as controls. The dogs were evaluated over a 6-month period using a range of outcome measures required for preclinical animal models, and it was shown that the use of internal brace for ACL reconstruction could effectively contribute to knee stability and improve function without causing premature osteoarthritis.

##### Anchors Primary ACL Repair

As it was noted about internal brace, only one animal study was found, after our search, about anchors primary ACL repair. Also, no cadaveric studies were found. The animal study by Seitz et al. comprised 20 sheep, which were randomly assigned to non-augmented primary ACL repair, or to augmented ACL repair with the use of a polyethylene terephthalate (PET) band [30]. The study focused on the histological changes during a 1-year postoperative observation period regarding necrosis and the loss of normal structures during the healing period. Histological healing was done after 16 weeks for the augmented repair group and after 26 weeks for the non-augmented repair group. It was shown that the use of polyethylene terephthalate (PET) band to augment ACL repair prevented the ligament from necrosis and loss of normal structures, in comparison with non-augmented repair group.

### Discussion

Till recently, the general opinion is that outcomes of open primary repair of the anterior cruciate ligament (ACL) in the historical literature were disappointing. But is that true? According to van der List et al., in a recent review of the old literature, tear location seems to have played a role on the outcomes of open primary ACL repair [26]. Outcomes of open primary repair in patients with proximal tears were excellent, which confirms that there may be a potential role for primary repair as treatment for proximal ACL tears [26].

More specifically, in another historical analysis by the same author, it was demonstrated that the open primary ACL repair was the most common treatment in the 1970s and 1980s, but because multiple studies noted deterioration of outcomes at mid-term follow-up, in addition to several randomized clinical trials (RCTs) that noted better outcomes following ACL reconstruction, the open primary repair technique was abandoned [25]. At the end of the open primary repair era, however, several studies showed that outcomes of open primary repair were good to excellent and did not deteriorate when this technique was selectively performed in patients with proximal ACL tears, whereas primary repair led to disappointing and unpredictable results in patients with mid-substance tears [25, 26]. Unfortunately, enrollment of patients in the aforementioned RCTs was already finished, ultimately leading to abandoning of open primary repair, despite the advantages of ligament preservation [25].

In 2005, Strand et al. in a long-term follow-up of open primary ACL repair suggested that open repair by suture is no longer recommended, while further research is indicated on the possible use of refixation of the ruptured ACL by arthroscopy [48]. Over the last few years, with the recognition of the importance of tear type and tissue quality, there has been a renewed interest in arthroscopic primary ACL repair [20, 24, 41, 43]. So, we noticed a crucial shift in favor of the preservation of the native ACL, even as a remnant in the cases of ACL reconstruction [49]. Kondo et al. showed that remnant preservation in anatomic double-bundle ACL reconstruction significantly improved postoperative knee stability [50]. The importance of proprioception in knee stability and self-protection from a re-rupture led the orthopedic science to investigate new primary ACL repair techniques [49, 50]. In a way, our findings suggest that orthopedic science has moved “back to the future” by rediscovering previous anterior cruciate ligament repair strategies couched in sophisticated, modernized surgical techniques of the twentieth-first century.

Nevertheless, a critical question emerges: why do we need primary ACL repair when we have well-established ACL reconstruction techniques? Indeed, the gold standard of ACL injuries remains nowadays the autograft reconstruction. However, many drawbacks of reconstructive surgery exist, such as anterior knee pain, muscle atrophy [51], and loss of range of motion [52]. Donor site pain affects 32–43% of patients after anterior cruciate ligament reconstruction when the autograft is freshly harvested bone-patellar tendon-bone tissue [53]. In addition, normal kinematics is not restored, and osteoarthritis is not prevented [54]. Finally, revision surgery, if necessary, can be devastating due to tunnel widening or tunnel malpositioning [52].

Particularly, abnormal joint motion has been linked to joint arthrosis after anterior cruciate ligament (ACL) reconstruction. The precise mechanisms contributing to joint arthrosis after ACL reconstruction are not well understood [46]. However, various factors have been suggested to contribute to the risk, ranging from the joint inflammatory response to altered biomechanics [55]. The altered rotational position could cause changes in tibiofemoral contact during walking that could cause the type of degenerative changes reported in the meniscus and the articular cartilage following ACL injury [54]. In addition, Ardern et al. illustrated that more than one fourth of the ACL-reconstructed patients were disappointed with the outcome [56]. Quadriceps atrophy is the most obvious remaining deficit after ACL reconstruction [51], while increased kneeling pain in the patellar tendon-reconstructed patients was seen consistently [57].

Finally, the tunnel widening after a re-ruptured ACL reconstruction or a possible mal-positioning of the tunnels might jeopardize the clinical outcome of these patients in a revision surgery [58]. On the other side, Kiapour et al. showed that despite nonanatomic positions of the femoral and tibial tunnels used in the bridge-enhanced repair procedure (tunnels drilled slightly anterior to the ACL footprint), repaired ACLs more closely replicated the vertical alignment of the contralateral intact ACL in the sagittal plane [46]. In contrast, the reconstructed grafts were substantially more vertical than their contralateral intact ACLs, even with anatomic tunnel positions. These observations indicate that the primary ACL repair is less sensitive to the tunnel positions and that repaired ACLs heal insertion to insertion [46].

According to the aforementioned arguments, there is still space to reconsider a possible maintenance of the native ACL, if there would be a possible evidence-based arthroscopic technique to support ACL primary biological repair. Towards this direction, four alternative operative techniques by using different medical technological devices have been proposed. This review focused on the existing published evidence in favor or against these methods.

**Fig. 4:**
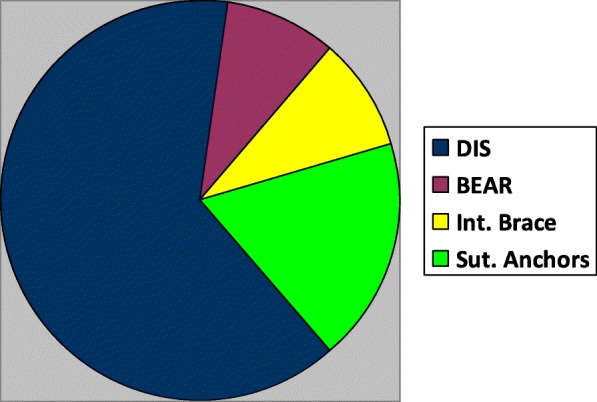
Pie chart illustrating the proportion of clinical studies included in the review that dealt with DIS, BEAR, internal brace and suture anchors. DIS: dynamic intraligamentary stabilization, BEAR: bridge-enhanced ACL repair, Int. Brace: internal brace, Sut. Anchors: suture anchors, ACL: anterior cruciate ligament

The majority (60%) of the published clinical trials concerning the primary ACL repair investigated the dynamic intraligamentary stabilization (DIS) with Ligamys, while only four studies (40% of the clinical trials) were found concerning the three other surgical techniques (Fig. 4).

According to Bieri et al., DIS has less working days loss than a conventional ACL reconstruction [31]. They conducted a post-hoc analysis of prospectively collected data in the Swiss National Accident Insurance Fund (SUVA) database, comparing 53 DIS patients with the same number of ACL reconstructed patients (ACLR). DIS patients benefited from nearly 1 month shorter absence from work than ACLR patients. This was the only comparative study we found in consideration of DIS.

Furthermore, another study of the same team suggested that 45 DIS-treated patients had successful functional recovery and low re-rupture rate at 1-year follow-up [38]. This follow-up period is considered to be rather small to evaluate this technique, while a control group of ACL-reconstructed patients, which was lacking in this study, might lead to safer conclusions. Even more, Eggli et al., in a pilot study of 10 patients treated with DIS, demonstrated a 5-year survival rate of 80% [39]. Two re-ruptures in 10 patients are in fact not a very low failure rate. The small number of patients as it was a pilot study is a major drawback of this survey, whereas the follow-up of 60 months is the longest that we documented among the DIS clinical studies.

Evangelopoulos et al. [40] dealt with the mid-substance tears which are controversial as a treatment category in primary ACL repair, while Kösters et al. [41], Henle et al. [20] and Eggli et al. [43] supported the use of microfractures around the femoral footprint of the native ACL in the acceleration of the healing-response. Henle et al. investigated the clinical outcome of DIS in a large case series of 278 patients [20]. Follow-up was up to 2 years, during which 11 patients were found with ACL re-rupture or insufficient subjective stability of the knee. Most patients exhibited almost normal knee function, reported excellent satisfaction, and were able to return to their previous levels of sports activity. Another interesting fact was that this strategy resulted in stable healing of all sutured menisci, which could lower the rate of osteoarthritic changes in future, according to the authors.

Furthermore, Häberli et al. in a biomechanical study of eight fresh-frozen human cadaveric knee joints showed that anteroposterior translation of knees treated with this dynamic augmentation procedure is low [45]. In another cadaveric study, it was shown that DIS with a preload of 80 N restores knee joint kinematics to a level comparable to that of an ACL-intact knee and is therefore capable of providing knee joint stability during ACL healing [35]. Finally, two non-clinical studies argued in favor of the use of DIS in future clinical trials [8, 27].

The BEAR in its current form may provide a valuable therapeutic option for patients with ACL tear. It takes advantage of the biologic basis of wound healing in other tissues by providing a stable provisional scaffold which can immobilize autologous blood in the otherwise fluid wound site. This technique has shown promise in preclinical models. The BEAR scaffold was manufactured at Boston Children’s Hospital and completed all biocompatibility and sterility testing prior to use in the clinical study [36, 37]. The scaffold comprised extracellular matrix proteins, including collagen, that were obtained from bovine tissue [28].

Considering the BEAR procedure, there was only one clinical trial by Murray et al. [32]. The number of patients is rather very small to make conclusions in this cohort level II study, which is the first on humans. The similar results between the BEAR group (10 patients) and the ACL reconstructed group (10 patients) seem hopeful, but still we need more data from larger series to evaluate correctly this technique. In addition, another weak point of this study was the very short follow-up of only 3 months. On the contrary, six animal studies showed that the BEAR procedure is safe and effective for the treatment of a fresh ACL rupture in the pre-clinical level [24, 28, 36, 37, 47, 48].

According to Smith et al. [33], the temporary internal brace (Arthrex) might be a very interesting solution for children with open periarticular physes who experience an ACL rupture. Requirement for this treatment is an adequate ACL remnant, which permits direct repair. The major disadvantage of this study is that it includes only 3 patients in a 2-year follow-up with no comparison to other treatment options. The excellent results cannot be taken into serious consideration before a larger series will be published. This was the only clinical study that we found concerning the internal brace, so there was no trial in adult populations apart from a case report with excellent results [44]. In a preclinical canine model, Cook et al. illustrated that using a quadriceps tendon allograft with internal brace (QTIB) prevented early failure, allowed for direct, four-zone graft-to-bone healing, and functional graft remodeling while avoiding problems noted with use of all-synthetic grafts [29]. Despite that, the use of a quadriceps allograft in combination with the synthetic internal brace is totally different than the clinical study in children, where the internal brace device was used without any graft to improve self-healing of the native ACL.

Last but not least, in a level III case-control study, Achtnich et al. compared the results of 20 patients treated with proximal refixation of the ACL using knotless suture anchors (Arthrex) and microfracturing with 20 single-bundle ACL-reconstructed patients [34]. The big difference in this study was that the operation was performed within 6 weeks from the injury. The results were not statistically different, but still we noticed a 15% re-rupture rate in the anchors-treated group while none from the reconstructed control group had a re-rupture. DiFelice et al. illustrated in a level IV study that ten out of 11 patients had good subjective and clinical outcomes after ACL preservation surgery with suture anchors (Arthrex) at a minimum of 2 years and mean of 3.5 years’ follow-up [42]. The very small number of patients in this case series does not help us prove the efficacy of this treatment. Further clinical studies are needed.

In summary, preservation of the proprioception of the native ACL, without donor site morbidity and minimal cartilage damage are the key-points which led to the research and development of new primary ACL repair techniques. Most of the clinical studies (especially the DIS clinical studies) suggest that primary ACL repair should be done during the first 14–21 days after a proximal ACL rupture and not later. Till now, among the 4 different primary ACL repair techniques, the DIS is by far the one, which has been used most frequently in patients (Fig. 4). The clinical results of the DIS were found comparable with the ACL reconstruction, including a small number of complications in most studies (Table 3).

**Table 3:** Clinical studies: failure rate and a brief conclusion per study

Study	Technique	Failures	Brief conclusion
Bieri et al. [31]	DIS	5 DIS and 4 ACLR revisions due to traumatic re-injuries and one DIS revision due to chronic instability.	Acceptable treatment option for ACL rupture
Büchler et al. [38]	DIS	Three re-ruptures during the first postoperative year.	Low re-rupture rate, satisfactory functional recovery.
Eggli et al. [39]	DIS	Two re-ruptures at 5 months and 4.2 years after surgery.	Excellent outcomes and satisfaction of patients.
Evangelopoulos et al. [40]	DIS	Re-rupture with subsequent instability in 6 patients without collagen application, and extension loss in 11 patients.	Additional application of a collagen membrane on ACL superior to solitary DIS.
Kösters et al. [41]	DIS	-One traumatic re-rupture. -Two removals of the monoblock and arthroscopic arthrolysis due to restricted RoM.	DIS with microfracturing of notch could biomechanically and biologically promote self-healing of a ruptured ACL.
Henle et al. [20]	DIS	Eight re-ruptures of the ACL, 3 mechanical insufficiencies.	DIS with anatomical repositioning and microfracturing, resulted in clinically stable healing.
Murray et al. [32]	BEAR	No differences in effusion or pain, no failures by Lachman examination criteria.	Low rate of adverse reactions.
Smith et al. [33]	Internal brace	None	Satisfactory alternative to ACL reconstruction, where an adequate ACL remnant allows direct repair.
Achtnich et al. [34]	Suture anchors primary ACL repair	The failure rate was 15% in the ACL re-fixation group and 0% in the reconstruction group.	Re-fixation of the ACL is a feasible option in selected patients.
DiFelice et al. [42]	Suture anchors primary ACL repair	None	Short-term clinical success in carefully selected patients with proximal avulsion-type tears and excellent tissue quality.

*DIS* dynamic intraligamentary stabilization, *BEAR* bridge-enhanced ACL repair, *ACL* anterior cruciate ligament, *ACLR* anterior cruciate ligament reconstruction, *RoM* Range of Motion

Preoperative magnetic resonance imaging (MRI) is not always enough to prove if a rupture is proximal or not. That is why this must be evaluated arthroscopically, so that the decision of choosing either a primary ACL repair technique or an ACL reconstruction will be taken intra-operatively.

Finally, future studies may clarify if biologically enhanced ACL repair could result in better outcomes. Gantenbein et al. demonstrated that mesenchymal stem cells and collagen patches could promote healing when they are used in a non-loading ACL repair model [59]. The authors underlined that further research concerning mechanical loading models is needed so as to confirm the promising results of their study.

## Conclusions

Arthroscopic primary ACL repair might totally change the way that the orthopedic surgeon thinks about ACL treatment. It is not yet clear whether this will be the new reality or just another trend in orthopedic evolution.

Further clinical evidence is needed for the techniques of bridge-enhanced ACL repair, internal brace and suture anchors ACL refixation to support the existing animal and cadaveric biomechanical studies. The existed clinical trials are not enough to establish the use of those techniques in the ACL-ruptured patients. On the contrary, the Dynamic intraligamentary stabilization with Ligamys™ device demonstrated very promising results in different types of clinical studies. Notwithstanding, the DIS clinical studies were mostly conducted from researchers coming from the same medical center, so a need is recognized for more independent studies implemented in different centers.

## Abbreviations

ACL: Anterior cruciate ligament
ACLR: Anterior cruciate ligament reconstruction
BEAR: Bridge-enhanced repair
DIS: Dynamic intraligamentary stabilization
IKDC: International Knee Documentation Committee
MRI: Magnetic resonance imaging
PET: Polyethylene terephthalate
QTIB: Quadriceps tendon graft with internal brace
RoM: Range of motion
SUVA: Swiss Accident Insurance Fund

## References

[1] Eichler K, Hess S, Riguzzi M, Can U, Brugger U (2015). Impact evaluation of Swiss medical board reports on routine care in Switzerland: a case study of PSA screening and treatment for rupture of anterior cruciate ligament. Swiss Med Wkly.

[2] Mall NA, Chalmers PN, Moric M, Tanaka MJ, Cole BJ, Bach BR (2014). Incidence and trends of anterior cruciate ligament reconstruction in the United States. Am J Sports Med.

[3] Biau DJ, Tournoux C, Katsahian S, Schranz PJ, Nizard RS (2006). Bone-patellar tendon-bone autografts versus hamstring autografts for reconstruction of anterior cruciate ligament: meta-analysis. BMJ.

[4] Herrington L (2013). Functional outcome from anterior cruciate ligament surgery: a review. OA Orthop.

[5] Muaidi QI, Nicholson LL, Refshauge KM, Herbert RD, Maher CG (2007). Prognosis of conservatively managed anterior cruciate ligament injury: a systematic review. Sports Med.

[6] Reinhardt KR, Hammoud S, Bowers AL, Umunna BP, Cordasco FA (2012). Revision ACL reconstruction in skeletally mature athletes younger than 18 years. Clin Orthop Relat Res.

[7] Parkkari J, Pasanen K, Mattila VM, Kannus P, Rimpel A (2008). The risk for a cruciate ligament injury of the knee in adolescents and young adults: a population-based cohort study of 46 500 people with a 9 year follow-up. Br J Sports Med.

[8] Kohl S, Evangelopoulos DS, Kohlhof H, Hartel M, Bonel H, Henle P, von Rechenberg B, Eggli S (2013). Anterior crucial ligament rupture: self-healing through dynamic intraligamentary stabilization technique. Knee Surg Sports Traumatol Arthrosc.

[9] Shelbourne KD, Gray T, Haro M (2009). Incidence of subsequent injury to either knee within 5 years after anterior cruciate ligament reconstruction with patellar tendon autograft. Am J Sports Med.

[10] Paterno MV, Schmitt LC, Ford KR, Rauh MJ, Myer GD, Huang B (2010). Biomechanical measures during landing and postural stability predict second anterior cruciate ligament injury after anterior cruciate ligament reconstruction and return to sport. Am J Sports Med.

[11] Hui C, Salmon LJ, Kok A, Maeno S, Linklater J, Pinczewski LA (2011). Fifteen-year outcome of endoscopic anterior cruciate ligament reconstruction with patellar tendon autograft for “isolated” anterior cruciate ligament tear. Am J Sports Med.

[12] Leys T, Salmon L, Waller A, Linklater J, Pinczewski L (2012). Clinical results and risk factors for reinjury 15 years after anterior cruciate ligament reconstruction: a prospective study of hamstring and patellar tendon grafts. Am J Sports Med.

[13] Aglietti P, Giron F, Buzzi R, Biddau F, Sasso F (2004). Anterior cruciate ligament reconstruction: bone-patellar tendon-bone compared with double semitendinosus and gracilis tendon grafts—a prospective, randomized clinical trial. J Bone Joint Surg Am.

[14] Stengel D, Klufmöller F, Rademacher G, Mutze S, Bauwens K, Butenschön K (2009). Functional outcomes and health-related quality of life after robot-assisted anterior cruciate ligament reconstruction with patellar tendon grafts. Knee Surg Sports Traumatol Arthrosc.

[15] Streich NA, Barié A, Gotterbarm T, Keil M, Schmitt H (2010). Transphyseal reconstruction of the anterior cruciate ligament in prepubescent athletes. Knee Surg Sports Traumatol Arthrosc.

[16] West RV, Harner CD (2005). Graft selection in anterior cruciate ligament reconstruction. J Am Acad Orthop Surg.

[17] Biau DJ, Tournoux C, Katsahian S, Schranz P, Nizard R (2007). ACL reconstruction: a meta-analysis of functional scores. Clin Orthop Relat Res.

[18] Barrack RL, Skinner HB, Buckley SL (1989). Proprioception in the anterior cruciate deficient knee. Am J Sports Med.

[19] Jerosch J, Prymka M (1996). Knee joint proprioception in normal volunteers and patients with anterior cruciate ligament tears, taking special account of the effect of a knee bandage. Arch Orthop Trauma Surg.

[20] Henle P, Röder C, Perler G, Heitkemper S, Eggli S (2015). Dynamic Intraligamentary Stabilization (DIS) for treatment of acute anterior cruciate ligament ruptures: case series experience of the first three years. BMC Musculoskelet Disord.

[21] Murray MM, Spindler KP, Devin C, Snyder BS, Muller J, Takahashi M (2006). Use of a collagen-platelet rich plasma scaffold to stimulate healing of a central defect in the canine ACL. J Orthop Res.

[22] Murray MM, Spindler KP, Abreu E, Muller JA, Nedder A, Kelly M (2007). Collagen-platelet rich plasma hydrogel enhances primary repair of the porcine anterior cruciate ligament. J Orthop Res.

[23] Silva A, Sampaio R (2009). Anatomic ACL reconstruction: does the platelet-rich plasma accelerate tendon healing?. Knee Surg Sports Traumatol Arthrosc.

[24] Kiapour AM, Fleming BC, Murray MM (2015). Biomechanical outcomes of bridge enhanced anterior cruciate ligament repair are influenced by sex in a preclinical model. Clin Orthop Relat Res.

[25] van der List JP, DiFelice GS (2017). Primary repair of the anterior cruciate ligament: a paradigm shift. Surgeon.

[26] van der List JP, DiFelice GS (2017). Role of tear location on outcomes of open primary repair of the anterior cruciate ligament: a systematic review of historical studies. Knee.

[27] Kohl S, Evangelopoulos DS, Ahmad SS, Kohlhof H, Herrmann G, Bonel H (2014). Eggli S. A novel technique, dynamic intraligamentary stabilization creates optimal conditions for primary ACL healing: a preliminary biomechanical study. Knee.

[28] Proffen BL, Perrone GS, Fleming BC, Sieker JT, Kramer J, Hawes ML, Murray MM (2015). Effect of low-temperature ethylene oxide and electron beam sterilization on the in vitro and in vivo function of reconstituted extracellular matrix-derived scaffolds. J Biomater Appl.

[29] Cook JL, Smith P, Stannard JP, Pfeiffer F, Kuroki K, Bozynski CC, Cook CA (2017). Canine arthroscopic anterior cruciate ligament reconstruction model for study of synthetic augmentation of tendon allografts. J Knee Surg.

[30] Seitz H, Menth-Chiari WA, Lang S, Nau T (2008). Histological evaluation of the healing potential of the anterior cruciate ligament by means of augmented and non-augmented repair: an in vivo animal study. Knee Surg Sports Traumatol Arthrosc.

[31] Bieri KS, Scholz SM, Kohl S, Aghayev E, Staub LP (2017). Dynamic intraligamentary stabilization versus conventional ACL reconstruction: a matched study on return to work. Injury.

[32] Murray MM, Flutie BM, Kalish LA, Ecklund K, Fleming BC, Proffen BL, Micheli LJ (2016). The bridge-enhanced anterior cruciate ligament repair (BEAR) procedure: an early feasibility cohort study. Orthop J Sports Med.

[33] Smith JO, Yasen SK, Palmer HC, Lord BR, Britton EM, Wilson AJ (2016). Paediatric ACL repair reinforced with temporary internal bracing. Knee Surg Sports Traumatol Arthrosc.

[34] Achtnich A, Herbst E, Forkel P, Metzlaff S, Sprenker F, Imhoff AB, Petersen W (2016). Acute proximal anterior cruciate ligament tears: outcomes after arthroscopic suture anchor repair versus anatomic single-bundle reconstruction. Arthroscopy.

[35] Schliemann B, Lenschow S, Domnick C, Herbort M, Häberli J, Schulze M, Wähnert D, Raschke MJ, Kösters C (2017). Knee joint kinematics after dynamic intraligamentary stabilization: cadaveric study on a novel anterior cruciate ligament repair technique. Knee Surg Sports Traumatol Arthrosc.

[36] Biercevicz AM, Proffen BL, Murray MM, Walsh EG, Fleming BC (2015). T2* relaxometry and volume predict semi-quantitative histological scoring of an ACL bridge-enhanced primary repair in a porcine model. J Orthop Res.

[37] Proffen BL, Perrone GS, Fleming BC, Sieker JT, Kramer J, Hawes ML, Badger GJ, Murray MM (2015). Electron beam sterilization does not have a detrimental effect on the ability of extracellular matrix scaffolds to support in vivo ligament healing. J Orthop Res.

[38] Büchler L, Regli D, Evangelopoulos DS, Bieri K, Ahmad SS, Krismer A, Muller T, Kohl S (2016). Functional recovery following primary ACL repair with dynamic intraligamentary stabilization. Knee.

[39] Eggli S, Röder C, Perler G, Henle P (2016). Five year results of the first ten ACL patients treated with dynamic intraligamentary stabilisation. BMC Musculoskelet Disord.

[40] Evangelopoulos DS, Kohl S, Schwienbacher S, Gantenbein B, Exadaktylos A, Ahmad SS (2017). Collagen application reduces complication rates of mid-substance ACL tears treated with dynamic intraligamentary stabilization. Knee Surg Sports Traumatol Arthrosc.

[41] Kösters C, Herbort M, Schliemann B, Raschke MJ, Lenschow S (2015). Dynamic intraligamentary stabilization of the anterior cruciate ligament. Operative technique and short-term clinical results. Unfallchirurg.

[42] DiFelice GS, Villegas C, Taylor S (2015). Anterior cruciate ligament preservation: early results of a novel arthroscopic technique for suture anchor primary anterior cruciate ligament repair. Arthroscopy.

[43] Eggli S, Kohlhof H, Zumstein M, Henle P, Hartel M, Evangelopoulos DS, Bonel H, Kohl S (2015). Dynamic intraligamentary stabilization: novel technique for preserving the ruptured ACL. Knee Surg Sports Traumatol Arthrosc.

[44] Wilson WT, Hopper GP, Byrne PA, MacKay GM (2016). Anterior cruciate ligament repair with internal brace ligament augmentation. Surg Technol Int.

[45] Häberli J, Henle P, Acklin YP, Zderic I, Gueorguiev B (2016). Knee joint kinematics with dynamic augmentation of primary anterior cruciate ligament repair - a biomechanical study. J Exp Orthop.

[46] Kiapour AM, Fleming BC, Murray MM (2017). Structural and anatomic restoration of the anterior cruciate ligament is associated with less cartilage damage 1 year after surgery: healing ligament properties affect cartilage damage. Orthop J Sports Med.

[47] Fleming BC, Proffen BL, Vavken P, Shalvoy MR, Machan JT, Murray MM (2015). Increased platelet concentration does not improve functional graft healing in bio-enhanced ACL reconstruction. Knee Surg Sports Traumatol Arthrosc.

[48] Strand T, Mølster A, Hordvik M, Krukhaug Y (2005). Long-term follow-up after primary repair of the anterior cruciate ligament: clinical and radiological evaluation 15-23 years postoperatively. Arch Orthop Trauma Surg.

[49] Andonovski A, Topuzovska S, Samardziski M, Bozinovski Z, Andonovska B, Temelkovski Z (2017). The influence of anterior cruciate ligament remnant on postoperative clinical results in patients with remnant preserving anterior cruciate ligament reconstruction. Open Access Maced J Med Sci.

[50] Kondo E, Yasuda K, Onodera J, Kawaguchi Y, Kitamura N (2015). Effects of remnant tissue preservation on clinical and arthroscopic results after anatomic double-bundle anterior cruciate ligament reconstruction. Am J Sports Med.

[51] Lindström M, Strandberg S, Wredmark T, Felländer-Tsai L, Henriksson M (2013). Functional and muscle morphometric effects of ACL reconstruction. A prospective CT study with 1 year follow-up. Scand J Med Sci Sports.

[52] van der List JP, DiFelice GS (2016). Preservation of the anterior cruciate ligament: a treatment algorithm based on tear location and tissue quality. Am J Orthop (Belle Mead NJ).

[53] Schandl K, Horváthy DB, Doros A (2016). Bone-albumin filling decreases donor site morbidity and enhances bone formation after anterior cruciate ligament reconstruction with bone-patellar tendon-bone autografts. Int Orthop.

[54] Andriacchi TP, Dyrby CO (2005). Interactions between kinematics and loading during walking for the normal and ACL deficient knee. J Biomech.

[55] Chu CR, Andriacchi TP (2015). Dance between biology, mechanics, and structure: a systems-based approach to developing osteoarthritis prevention strategies. J Orthop Res.

[56] Ardern CL, Österberg A, Sonesson S, Gauffin H, Webster KE, Kvist J (2016). Satisfaction with knee function after primary anterior cruciate ligament reconstruction is associated with self-efficacy, quality of life, and returning to the preinjury physical activity. Arthroscopy.

[57] Spindler KP, Kuhn JE, Freedman KB, Matthews CE, Dittus RS, Harrell FE (2004). Anterior cruciate ligament reconstruction autograft choice: bone-tendon-bone versus hamstring: does it really matter? A systematic review. Am J Sports Med.

[58] Maak TG, Voos JE, Wickiewicz TL, Warren RF (2010). Tunnel widening in revision anterior cruciate ligament reconstruction. J Am Acad Orthop Surg.

[59] Gantenbein B, Gadhari N, Chan SC, Kohl S, Ahmad SS (2015). Mesenchymal stem cells and collagen patches for anterior cruciate ligament repair. World J Stem Cells.

